# How UV light lowers the conductivity of SrTiO_3_ by photochemical water splitting at elevated temperature

**DOI:** 10.1039/d1ma00744k

**Published:** 2022-02-07

**Authors:** Alexander Viernstein, Markus Kubicek, Maximilian Morgenbesser, Tobias M. Huber, Matthäus Siebenhofer, Jürgen Fleig

**Affiliations:** Institute of Chemical Technologies and Analytics, TU Wien Getreidemarkt 9 164/EC 1060 Vienna Austria markus.kubicek@tuwien.ac.at; Centre of Electrochemical Surface Technology GmbH Viktor-Kaplan-Straße 2 2700 Wiener Neustadt Austria

## Abstract

Nominally undoped SrTiO_3_ single crystals were illuminated by UV light at 350 °C in oxidizing as well as reducing atmospheres. In N_2_/O_2_ atmospheres, UV irradiation enhances the conductivity of SrTiO_3_ by several orders of magnitude. In dry H_2_ atmosphere UV exposure leads to the opposite conductivity effect, *i.e.*, above band gap energy illumination surprisingly lowers the conductivity. This is discussed in the framework of a defect chemical model. We show that a shift in defect concentrations due to UV-driven oxygen incorporation from the gas phase into the oxide is the main cause of the measured conductivity changes. A model is introduced to illustrate the thermodynamic and kinetic drivers of the processes under UV irradiation. Noteably, in reducing H_2_/H_2_O atmospheres, the incorporation of oxygen into the investigated oxide under UV light takes place *via* water splitting. Owing to the predominant electron conduction of SrTiO_3_ in equilibrium with H_2_, oxygen incorporation upon UV and thus an increase of the oxygen chemical potential leads to a decrease of the majority electronic charge carrier, here electrons, which lowers the conductivity under UV irradiation.

## Abstract 2

Explaining the impact of oxygen incorporated into SrTiO_3_ single crystals in strongly reducing H_2_/H_2_O containing atmospheres under UV illumination at 350 °C.

## Graphical abstract

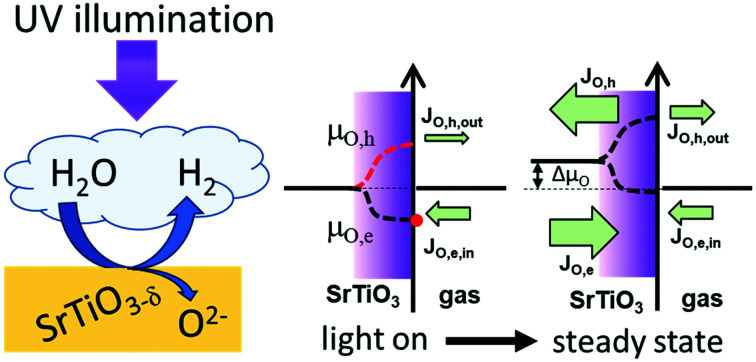

## Introduction

In recent years, the properties of the model perovskite SrTiO_3_^1–6^ upon above band gap light exposure at elevated temperatures came into the focus of research. Several different phenomena were observed and described, such as UV-induced changes of the oxygen exchange kinetics,^7^ light induced battery type voltages in solid oxide cells,^8^ varying photovoltages in SrTiO_3_ based high temperature solar cells,^9^ high temperature photochromism,^10^ and conductivity variations under UV illumination.^8,11,12^ Many of these observations can be explained by assuming a light driven oxygen incorporation into SrTiO_3_ and an increase of the oxygen chemical potential in the entire SrTiO_3_ bulk.^10^ However, in literature also the formation of oxygen vacancies as a consequence of UV illumination is described.^12^ Even though different UV-driven defect chemical consequences are not necessarily in contradiction to each other, questions about the factors determining the defect chemical situation under UV irradiation arise from the mentioned findings. Thus, experiments in a large *p*_O_2__ range are of high interest.

In this contribution, we show the effect of UV light on the conductivity of undoped SrTiO_3_ single crystals for four different gas atmospheres with very different oxygen partial pressures (air, N_2_ with some residual oxygen, and dry or humidified H_2_) with special emphasis on measurements under reducing conductions. The results are compared with the defect chemical predictions of a recently developed defect chemical model of the same type of SrTiO_3_ single crystals.^13^ In dry H_2_, measurements reveal that UV light lowers the bulk conductivity of SrTiO_3_, which is extraordinary, due to the unavoidable contributions of photoconductivity. It indicates that oxygen is driven into the crystal under UV irradiation even in H_2_/H_2_O atmosphere. There, however, the oxygen to be incorporated stems from water molecules and the measured conductivity is inseparably connected to UV-induced water splitting at elevated temperature, namely 350 °C.

## Experimental

In this study undoped (001) oriented SrTiO_3_ single crystals (5 × 5 × 0.5 mm^3^, CrysTec GmbH, Germany) which are indeed slightly p-type due to cation vacancies from growth were used. A detailed investigation on the same batch of single crystals revealed that both charge-neutral defects as well as defect-chemically relevant defects acting as acceptor, donor, or electron/hole trap states are present. The most important charge-neutral defects found were Ca(Sr) (<47 ppm), Ba(Sr) (1.8 ± 0.2 ppm), the most important charged defect were cation vacancies, most probably Ti-vacancies (6 ppm), additionally Al(Ti) (<0.2 ppm), and another yet unidentified (most probably associate-type) defect in similar sub-ppm concentration as Al(Ti) were found.^13^ In order to prepare the specimens for the in-plane impedance measurements, they were cleaned with ethanol in an ultrasonic bath, subsequently they were annealed for 12 h at 900 °C in air to reduce surface defects and afterwards cleaned again as above. Then, the samples were equilibrated at 700 °C either in air, N_2_ (containing approx. 70 ppm O_2_), humidified H_2_, or dry H_2_. The strongly reducing atmospheres containing H_2_ were established using commercially available gas mixtures of Ar and 2% of H_2_ (Messer, Germany). For humidification, the gas was passed through double-distilled water at room temperature. After annealing, the temperature was lowered to the actual measurement temperature of 350 °C while keeping the atmosphere, and the single crystals were again equilibrated at least for 12 h. Owing to this comparatively low temperature some deviations from equilibrium defect concentrations at 350 °C might still be present. Then the actual electrical measurements were performed.

**Fig. 1:**
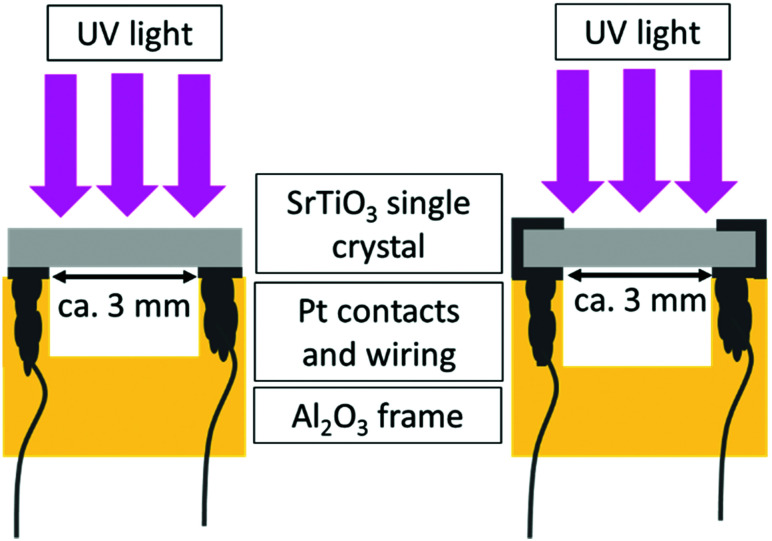
Sketches of the impedance spectroscopy set-up used in this study; left: The SrTiO_3_ single crystal is only contacted on the back side; right: Pt contacts are on both the front and back side. The specimens are connected directly to the impedance analyzer *via* Pt.

**Fig. 2:**
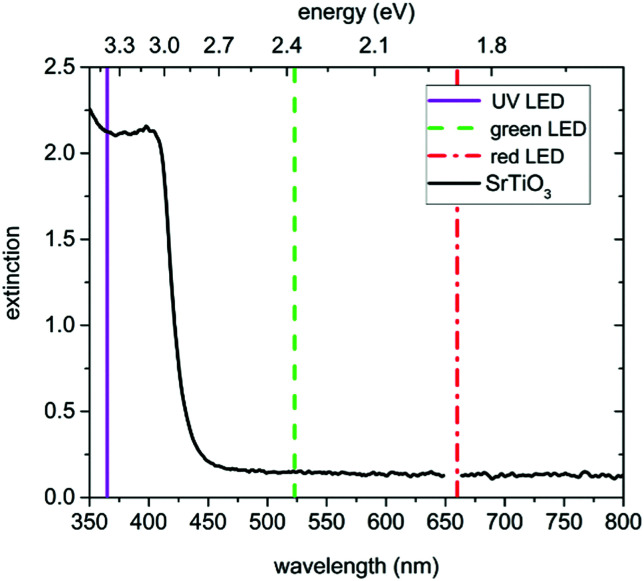
Extinction spectrum of a both side polished SrTiO_3_ single crystal at 350 °C in air. Additionally, the emitted wavelengths of the used UV (365 nm), green (523 nm), and red (660 nm) LEDs are indicated.

The conductivity before, under and after UV illumination was measured by impedance spectroscopy in an in-plane electrode geometry using Pt paste electrodes applied either only on the back side or on both the back and the front side of the samples (see Fig. 1). For comparison, also illumination by green and red light was performed. A flame polished quartz rod acted as light guide for the UV (365 nm), green (523 nm), and red (660 nm) light emitted by high performance LEDs (LED Engin, USA). The irradiation of SrTiO_3_ with UV light causes the excitation of electrons from the valence band to the conduction band and consequently leads to the formation of electron–hole pairs. Green and red light are not absorbed by SrTiO_3_ (compare absorption spectrum in Fig. 2) at 350 °C.

In-plane impedance spectra were recorded at 350 °C and between 1 MHz and 1 Hz using Alpha-A High Performance Analyzers (Novocontrol Technologies, Germany).

## Results

The in-plane conductivities of undoped SrTiO_3_ single crystals were deduced from the impedance spectra measured before, under, and after UV illumination. In air as well as in N_2_ we found a dominating high frequency arc (representing the bulk resistance and geometrical capacitance) and some low frequency features due to the electrodes.^6,11,14–16^ In dry and humidified H_2_, only one almost ideal semicircle was obtained in the investigated frequency range. Based on their capacitance values the dominating arcs can be attributed to SrTiO_3_ bulk and were fitted to a resistor in parallel to a constant phase element using Zview (Scribner Associated Inc.). In-plane sample conductivities were derived from the resistance assuming 1D current flow (length approx. 3 mm) and thus neglecting edge effects due to the exact electrode geometry.

**Fig. 3:**
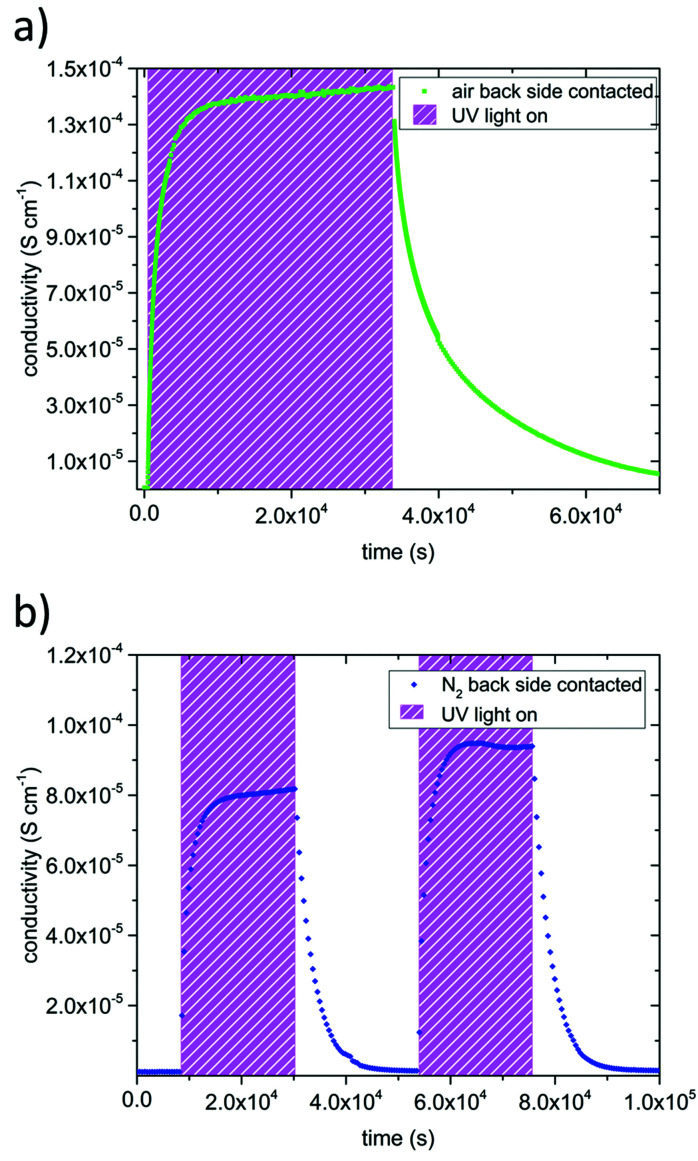
In-plane conductivities of back side contacted SrTiO_3_ before, under, and after UV irradiation in air (a) and N_2_ (b) at 350 °C. During UV illumination, the conductivity increased by orders of magnitude. After the UV light was turned off, several hours were needed before the initial conductivity was restored.

**Fig. 4:**
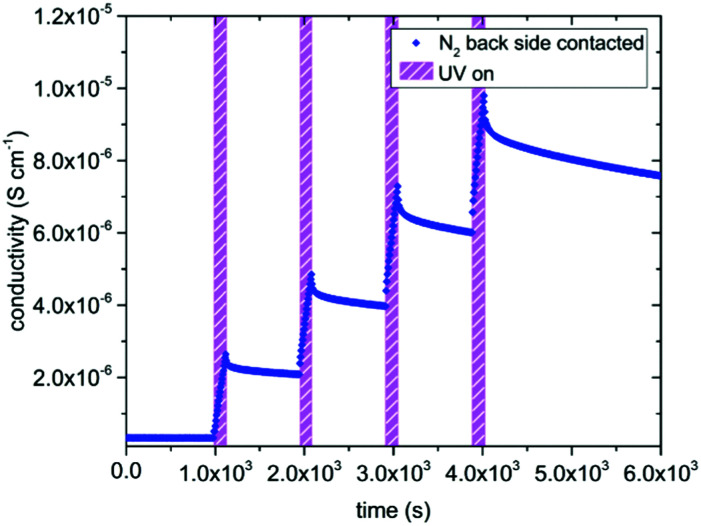
Due to the large time constant of the relaxation after the UV light was turned off, the in-plane conductivity increased more and more during each of the four short illumination periods (136 s each) at 350 °C in N_2_. In total the in-plane conductivity was increased by a factor of 30.

Fig. 3 displays results of measurement cycles in air and N_2_ during which the specimens were illuminated by UV light for several hours. The results are very similar, with drastic conductivity increases (up to more than two orders of magnitude) under UV irradiation on a time scale of about an hour. However, the maximum conductivities were lower in N_2_ than in air. The conductivity remained very high right after the UV light was switched off and then slowly relaxed on the time scales of several hours. The relaxation was slower in air than in N_2_. These extended relaxation time scales also led to the phenomenon that the conductivity could be driven up to higher values by subsequent short illumination periods (136 s) and longer dark times (832 s) as shown for N_2_ in Fig. 4. Before UV irradiation, an in-plane conductivity of 3.3 × 10^−7^ S cm^−1^ was measured and driven up to 2.6 × 10^−6^ S cm^−1^ during the first illumination period. This increase was followed by a minimal conductivity decrease to 2.1 × 10^−6^ S cm^−1^ after the UV source was turned off for the first time. Finally, after the fourth and last UV irradiation of SrTiO_3_, its conductivity reached almost 10^−5^ S cm^−1^, which is in total a conductivity increase by a factor of nearly 30. Irradiation with red or green light led only to a very minor conductivity increase, probably due to a small temperature increase when the light was absorbed by the Pt paste and/or corundum frame.

**Fig. 5:**
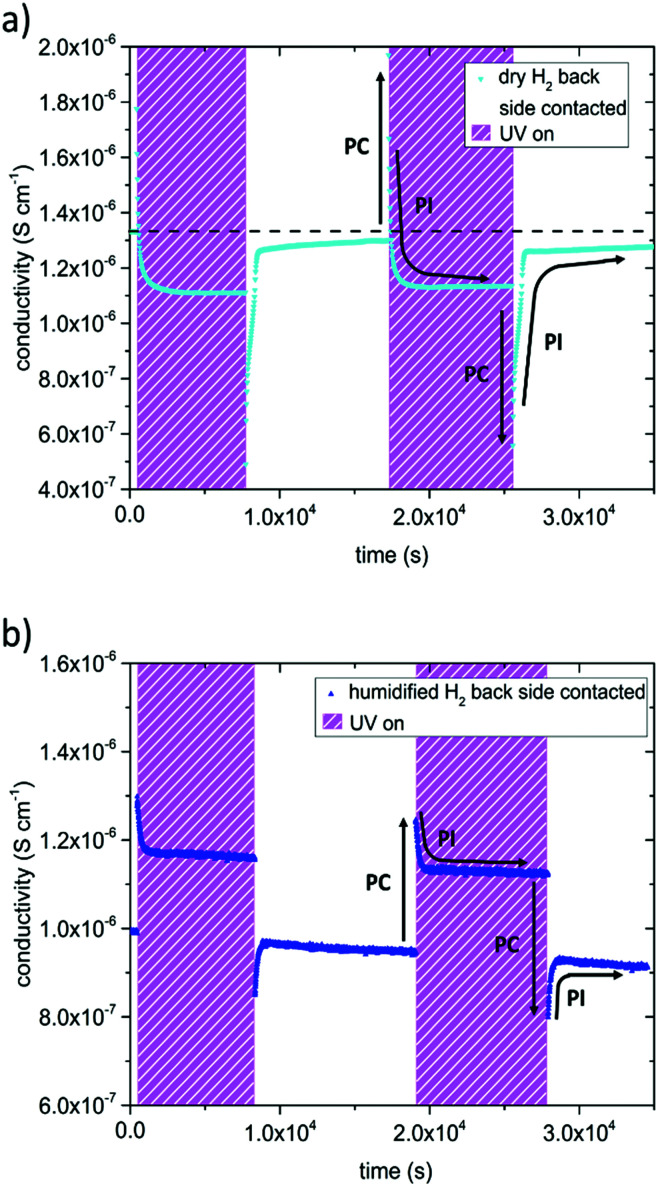
Results of impedance measurements performed in dry H_2_ (a) and humidified H_2_ (b) at 350 °C. The samples were contacted on the back side only. UV light was turned on twice leading to an abrupt conductivity increase due to photoconductivity (symbolized by black arrows marked with PC) followed by a steady decline of the conductivity (photo-ionic effect (PI)). In dry H_2_ even a plateau below the initial value was found. After the UV light was turned off, conductivity dropped promptly, then increased and approached the initial value.

In dry and humidified H_2_ at 350 °C the SrTiO_3_ sample is in the electron conducting regime.^13^ UV induced effects are still present but several differences were found compared to the studies in O_2_ containing gases (Fig. 5): (i) UV induced effects took place much faster. (ii) They were much less pronounced. (iii) UV may not only increase but also decrease the conductivity. More specific, the in-plane conductivity of a back side contacted SrTiO_3_ single crystal instantly jumped from 1.3 × 10^−6^ S cm^−1^ to 1.8 × 10^−6^ S cm^−1^ in the first and 2.0 × 10^−6^ S cm^−1^ in the second cycle (cyan triangle Fig. 5a) after the UV light was turned on. It is discussed in more detail below that the instant effect was caused by additional photoconductivity due to electron–hole formation upon UV. This increase became counter-balanced by a moderately fast decrease of the conductivity within a few 100 s until a reproducible plateau at 1.1 × 10^−6^ S cm^−1^ was established, which was approx. 15% below the original conductivity. After switching the UV light off, the conductivity dropped to 4.9 × 10^−7^ S cm^−1^ and to 5.6 × 10^−7^ S cm^−1^, respectively, *i.e.* less than 50% of the original value, due to the loss of photoconductivity. This immediate decline was followed by a conductivity increase within a few 100 s and finally nearly the initial value before illumination was again reached. Thus, two processes namely the fast-evolving photoconductivity (PC) and a slower photo-ionic (PI) process determined the evolution of the sample conductivity under reducing conditions. A detailed discussion of the photo-ionic effects affecting the entire sample is given below.

A very similar picture resulted for the experiments on back side contacted specimens conducted in humidified H_2_ atmosphere (compare Fig. 5b). Since the *p*_O_2__ was three to four orders of magnitude higher compared to dry H_2_, the initial conductivity of the electron conducting SrTiO_3_ was lower, and changes were not as distinct. The measurements started at a conductivity of 9.9 × 10^−7^ S cm^−1^, when UV light was turned on it peaked at 1.3 × 10^−6^ S cm^−1^ (1.2 × 10^−6^ S cm^−1^ respectively), and finally reached 1.2 × 10^−6^ S cm^−1^ (1.1 × 10^−6^ S cm^−1^) under UV illumination. After switching the UV light off, the conductivity dropped to 8.5 × 10^−7^ S cm^−1^ (8.0 × 10^−7^ S cm^−1^) and then relaxed to approximately the initial value.

In the following the differences between only back side and both-side contacted samples are shown. Here we consider the inverse resistance (1/*R* = conductance) of both measurement configurations and for the sake of comparability, the curves were normalized to the initial 1/*R* values before UV illumination. This eliminates differences in the electrode geometry, defect chemistry due to slow pre-equilibration or atmosphere and temperature.

**Fig. 6:**
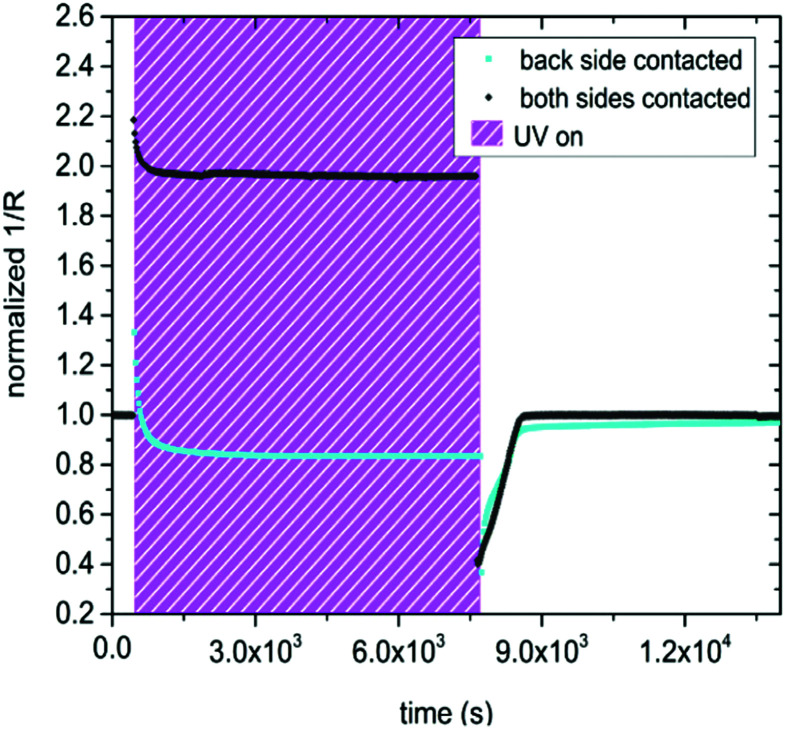
Inverse resistance (conductance) of a sample only contacted on the back side (orange squares) and a specimen contacted on both the back and the front side (black diamonds). The data were obtained in dry H_2_ at 350 °C. Both curves were normalized to the initial inverse resistance (before UV) for the sake of comparability. The initial photoconductivity related jump is more distinct in the both-side contacted sample. During irradiation both specimens showed a contrasting behavior, due to the different emphasis of the illuminated surface region. After the UV light was turned off the inverse resistance dropped to 40% of the initial (before UV irradiation) values in both cases.

Contacting the sample on both the front and the back side, led to a substantial change in the observed effect of UV exposure, compared to specimens contacted only on the back side, see Fig. 6. Due to photoconductivity, the inverse resistance of a both-side contacted SrTiO_3_ single crystal immediately jumped up after UV light was turned on. The jump was much more pronounced for the both-side contacted sample (by a factor of 2.2) compared to the only back side contacted SrTiO_3_ specimen (by a factor of 1.3). In contrast to the results shown in Fig. 5a the inverse resistance remained far above the initial value for contacts on both sides, and only a small decline by approx. 11% compared to the inverse resistance maximum was detectable. After the UV light was switched off, the inverse resistance of the both-side contacted specimen drastically dropped to approx. 40% of the initial value (PC) and finally relaxed back to the amount obtained before UV irradiation (PI), in accordance with the only back side contacted sample. Also, both specimens seem to exhibit very similar time constants, since the conductivity plateau under UV irradiation as well as the relaxation to the initial value after UV illumination were established nearly after the same time. This suggest that the fast photoconductivity effects were different, while the slower photo-ionic effects were the same. This is not surprising since PC effects take place primarily in the absorption zone close to the surface.

## Discussion

### Quantitative comparison with the defect chemical model of undoped SrTiO_3_

**Fig. 7:**
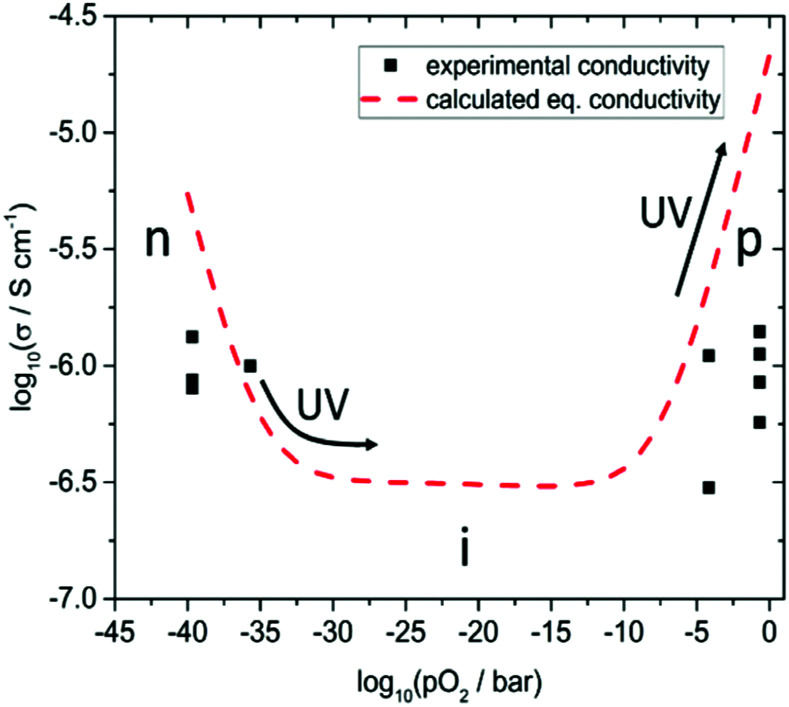
Measured in-plane conductivity (black squares) after heating the specimens to 700 °C and subsequently annealing at 350 °C for several hours in dry H_2_, humidified H_2_, N_2_, and air. Calculated conductivity assuming 6 ppm Ti vacancies as predominant dopant (broken red line) at 350 °C *vs. p*_O_2__, to give an impression of the change from p-type (p) conductivity at high oxygen partial pressures, to ionic (i), and finally to n-type (n) conductivity the lower *p*_O_2__ becomes. Arrows indicate stoichiometry changes in the bulk caused by UV.

In the following, we discuss the UV light induced changes found in nominally undoped SrTiO_3_ in more detail. To do so, we start with the conductivity values measured in dark for the different atmospheres. Fig. 7 shows several experimental results (black squares) of various in-plane conductivity measurements obtained in the four described atmospheres at 350 °C before UV illumination.

These data are compared with calculated conductivities derived from a detailed defect chemical model recently established for such undoped SrTiO_3_ single crystals. Among others, the defect model is based on impedance measurements in a wide *p*_O_2__ range between 500 and 700 °C.^13^ At these temperatures an equilibration with the gas phase takes place on a reasonable time scale and thus it was possible to extract equilibrium conductivities and chemical capacitances, which both lay the foundation of an extended defect model.^13^ Essentially, it was shown that cation vacancies, most probably approx. 6 ppm Ti vacancies cause a slight p-type doping in the considered nominally undoped SrTiO_3_ single crystals. From this data set, we can also predict the equilibrium conductivities for other *p*_O_2__ and temperatures. For 350 °C we found that hole conductivity dominates above a *p*_O_2__ of approx. 10^−8^ bar, electron conduction below 10^−32^ bar and in between a broad range with constant ionic conductivity due to the oxygen vacancies balancing the acceptor doping, see Fig. 7.

While the conductivity determined in humidified H_2_ fits excellently to the prediction, measured values are somewhat too small in dry H_2_, air, and N_2_. Moreover, those values differ from experiment to experiment, *i.e.* they depend on the exact prehistory, indicating that equilibrium with the gas phase is hardly well-established at 350 °C, despite all specimens were first annealed at 700 °C and subsequently equilibrated for several hours at 350 °C in the respective atmosphere. This is in accordance with the statements that in SrTiO_3_ oxygen surface exchange reactions are slow or even frozen-in at lower temperatures.^17,18^ Many days or even weeks might have been required for complete equilibration. Equilibrium values, however, seem to be reached in humidified H_2_ at 350 °C, suggesting that the corresponding gas exchange reaction1
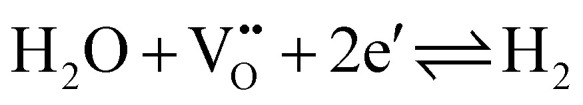
is still fast enough to establish thermodynamic equilibrium here. These somewhat ill-defined defect chemical states of several samples, however, do not interfere with all the following interpretations and conclusions.

### Attributing the UV driven conductivity changes to processes in SrTiO_3_

The time scales on which the conductivity changes under UV irradiation in all four atmospheres are by far too large to be only caused by photoconductivity, *e.g.* by the formation or recombination of electron–holes in the illuminated zone of the SrTiO_3_ single crystal. In Cr-doped SrTiO_3_ such recombination processes take place mostly within approx. 9 μs at room temperature.^19^ We assume similar time constants in undoped SrTiO_3_. Furthermore, the used UV light is mostly absorbed approx. within the first few μm.^9,20^ Thus, the in-plane conductivity changes of all samples cannot be explained by photoconductivity. This is true for the drastic increases of back side contacted specimens by more than (nearly) two orders of magnitude in air (N_2_), and also for the (somewhat faster) decrease of the conductivity in dry hydrogen. In these cases, the whole bulk has to be affected by the UV illumination *via* changes of its defect chemistry.

In a detailed earlier study on Fe-doped SrTiO_3_, it was already shown that UV-driven oxygen incorporation into the entire bulk is responsible for severe long-term conductivity changes in air.^10^ A main reason is the acceleration of oxygen incorporation kinetics into SrTiO_3_ under UV light.^7^ The same conclusion was also drawn to explain UV induced voltage measured in an electrochemical cell consisting of undoped SrTiO_3_/yttria-stabilized-zirconia/Pt in air under UV.^8^ Accordingly, UV-induced oxygen stoichiometry changes in the entire bulk of SrTiO_3_ are assumed to be the reason also for the large conductivity increase in air and N_2_. Below, we discuss in more detail that the same effect is responsible for the conductivity decrease measured here in dry H_2_. In this discussion, key question to be addressed is whether the chemical potential of oxygen in the bulk of SrTiO_3_ increases or decreases under UV illumination. Here, Fig. 7 gives a clear indication for the measurements in air and N_2_ only an increase of the oxygen chemical potential can explain the huge increase of conductivity found in our studies. In air, a nominal *p*_O_2__ increase of up to about seven orders of magnitude is found, which is also in accordance with electrochemical (battery-type) voltages measured on similar crystals with an ion-conducting bottom layer.^8^ Oxygen is driven into the samples and by filling oxygen vacancies holes are formed, which increase the conductivity in the entire bulk.^5,21–24^ The time dependence is governed by the oxygen chemical diffusion coefficient as shown in ref. 10 and as also will be detailed for undoped SrTiO_3_ in a forthcoming paper.

In the following, we concentrate on the remarkable fact that UV irradiation of SrTiO_3_ single crystals leads to a reduced in-plane conductivity in dry H_2_. As already mentioned above, the instant conductivity increase (decrease) observed in H_2_ when the UV light was turned on (off) are largely due to photo-conductivity (PC). It is manifest to assume that the slower change to lower values upon UV (PI), seen in H_2_, is the eqivalent to what we also see in air and N_2_, namely the change of the conductivity due to an oxygen stoichiometry changes in the bulk. From the conductivity *vs. p*_O2_ curve in Fig. 7 we can thus again conclude whether oxygen incorporation or oxygen release takes place under UV. Since at such low *p*_O2_ SrTiO_3_ is a n-type semiconductor only the filling of oxygen vacancies and the formation of holes (*i.e.* annihilation of electrons) can explain the observed lowered conductivity. Hence, like in air and N_2_, also in H_2_ UV pumps the crystal to a higher oxygen chemical potential. When the UV illumination stops, an instant drop of the conductivity occurs (PC) and only the photo-ionically decreased bulk conductivity determines the impedance of the specimen which then slowly relaxes by oxygen release. The decrease of the conductivity in H_2_ due to UV-triggered oxygen incorporation is much less pronounced than the increase measured in air. This can be easily understood from Fig. 7: the conductivity cannot drop beneath the ionic conductivity plateau. Most probably this plateau was reached in our experiments, and we cannot estimate the effective *p*_O2_ change in SrTiO_3_ from the measured conductivity.

This interpretation is also supported by the second measurement mode, *i.e.* with single crystals being contacted on both the illuminated front and the dark back side. Here the fast increase of the inverse resistance right after the UV light was turned on was more pronounced and the slow decrease of the inverse resistance under UV illumination was by far smaller than if the specimens were only contacted on the back side (see Fig. 6). However, shortly after the UV irradiation the inverse resistance reached approx. 40% of the initial value in both experimental configurations. This is in perfect accordance with our interpretation of a thin top surface region where the UV light is absorbed, and where the conductivity is strongly enhanced due to the photo generation of charge carriers. Below this zone the bulk conductivity is decreased due to a change in the oxygen content/chemical potential. Large parts of the bulk seem to be involved since otherwise an effective conductivity (or conductance) decrease of the entire sample can hardly be explained from the given conductivity *p*_O_2__ diagram. Thus, when electrodes are placed on the illuminated front side, the UV absorption zone becomes more decisive for the overall in-plane resistance (or conductance) of the investigated sample. In the following section we estimate the current density and the potential distribution in the two regions using finite element calculations.

The question remains, where the incorporated oxygen comes from when SrTiO_3_ single crystals are under UV illumination in strongly reducing H_2_ containing atmosphere. The oxygen stems from water, *i.e.* we face water splitting under UV light and thus hydrogen production rather than oxygen reduction. Accordingly, we suggest that UV illumination of SrTiO_3_ in H_2_ enhances the water splitting rate (including the corresponding oxygen incorporation into SrTiO_3_) without enhancing the reverse reaction in the same manner. This UV effect is not surprising since SrTiO_3_ is a well-known photocatalyst for water splitting.^25–28^

### Conductivity analysis and current distribution

In accordance with our model introduced in the last section, we estimated the conductivity of the bulk before UV illumination, the photoconductivity in the first μm of the illuminated specimen, and the decreased bulk conductivity under UV light for a SrTiO_3_ single crystal contacted on both the front and the back side. To do so, a parallel circuit was considered consisting of two resistors representing the bulk (*R*_bulk_) and the UV absorption region (*R*_surf_). We assume that the bulk does not change significantly during the first five to ten seconds of UV exposure. From the impedance of the first data point (*R*_UV_) after the UV light was switched on and the in-plane resistance before UV irradiation (*R*_bulk,dark_), the resistance of the thin UV region (*R*_surf_) can be calculated. According to2
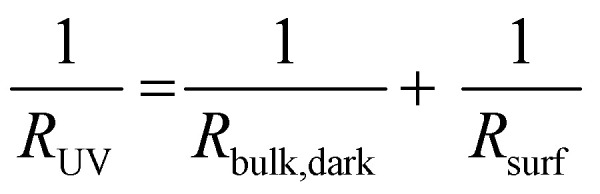
and assuming a thickness of 1 μm in which the UV light is mostly (approx. 80%)^9^ absorbed, the effective photoconductivity in this 1 μm layer is by a factor of approx. 600 higher than the bulk conductivity before UV irradiation. Shortly after the UV illumination the overall in-plane conductivity declined to 40% of the initial value, indicating that R_bulk_ had increased upon UV by a factor of 2.5.

**Fig. 8:**
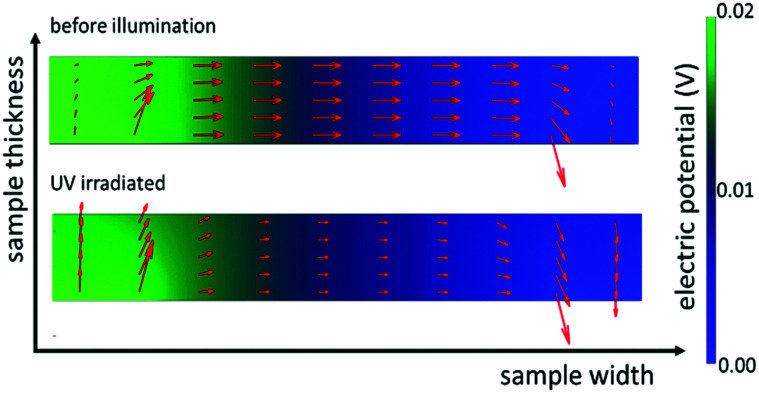
Finite element simulations of back side contacted SrTiO_3_ before UV illumination (top) and under UV irradiation in the steady state case (bottom). The red arrows indicate the direction and amount of current flowing from the one electrode (left side) to the other (right side). The thin UV illuminated region is not visible in this sketch. Additionally, the electric potential over the sample is indicated by the color bar on the right.

**Fig. 9:**
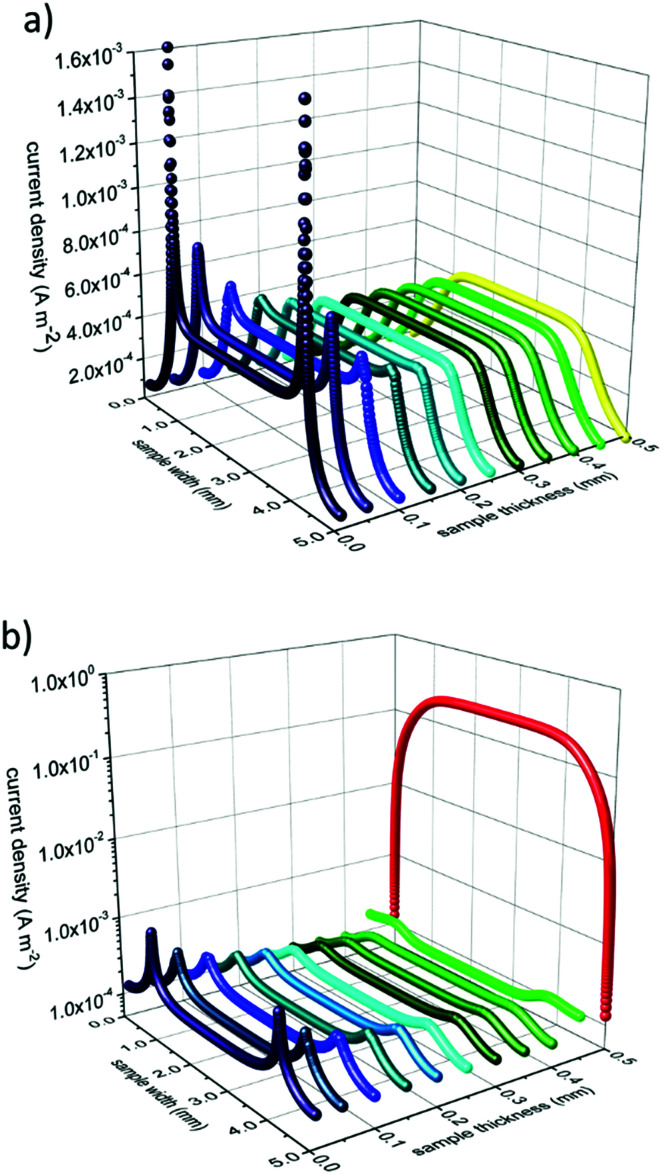
Current density before (a) and under UV illumination (b) of a back side contacted sample. The front side, illuminated by UV light, is at 0.5 mm sample thickness, the contacted back side at 0 mm. Current density is strongly enhanced in the UV absorption zone (red balls) which is assumed to be 1 μm thick in the simulation. In the dark bulk the current density is slightly reduced under UV illumination compared to that in the bulk before UV light was turned on.

Subsequently we used these numbers to perform finite element simulations (compare Fig. 8). Assuming an applied voltage of 0.02 V between the two electrodes at the back side of the sample the potential distribution and current flow in the bulk before and during UV illumination can be calculated. Before illumination, the current density between the two electrodes is nearly uniformly distributed over the whole thickness of the sample. During UV irradiation the current density is decreased in the dark bulk and strongly increased (by more than three orders of magnitude) in the UV region (see Fig. 9).

These simulations graphically show the impact of the formation of a zone with an enhanced photoconductivity and of a bulk region exhibiting a lower conductivity due to oxygen incorporation and diffusion. Additionally, it becomes more evident that the differing experimental results in Fig. 6 are caused by the differences in the measurement configurations and support our interpretation.

### Quantitative analysis of the time dependency of the conductivity changes

The time dependent changes of undoped SrTiO_3_ in air upon UV can be described by the equation known from diffusion-limited conductivity relaxation and we can derive a chemical diffusion coefficient of 6.3 × 10^−7^ cm^2^ s^−1^ More details on this analysis, including temperature dependences will be given in a forthcoming paper. Here, we concentrate on the conductivities measured in nitrogen and hydrogen. Those time dependent conductivity changes under UV are better described by a conductivity relaxation model with surface limitation, *i.e.* by the eqn (3).^29–31^3
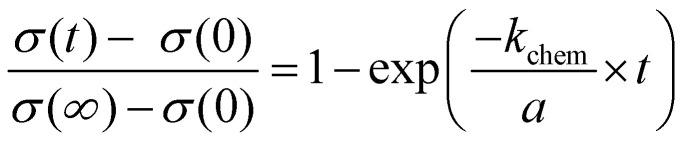


A constant oxygen chemical exchange coefficient *k*_chem_ is still assumed despite the large chemical potential changes considered here. The symbol *σ*(0) denotes the initial conductivity, *σ*(∞) the final conductivity, *a* is the sample thickness, and *t* the time of UV illumination.

**Fig. 10:**
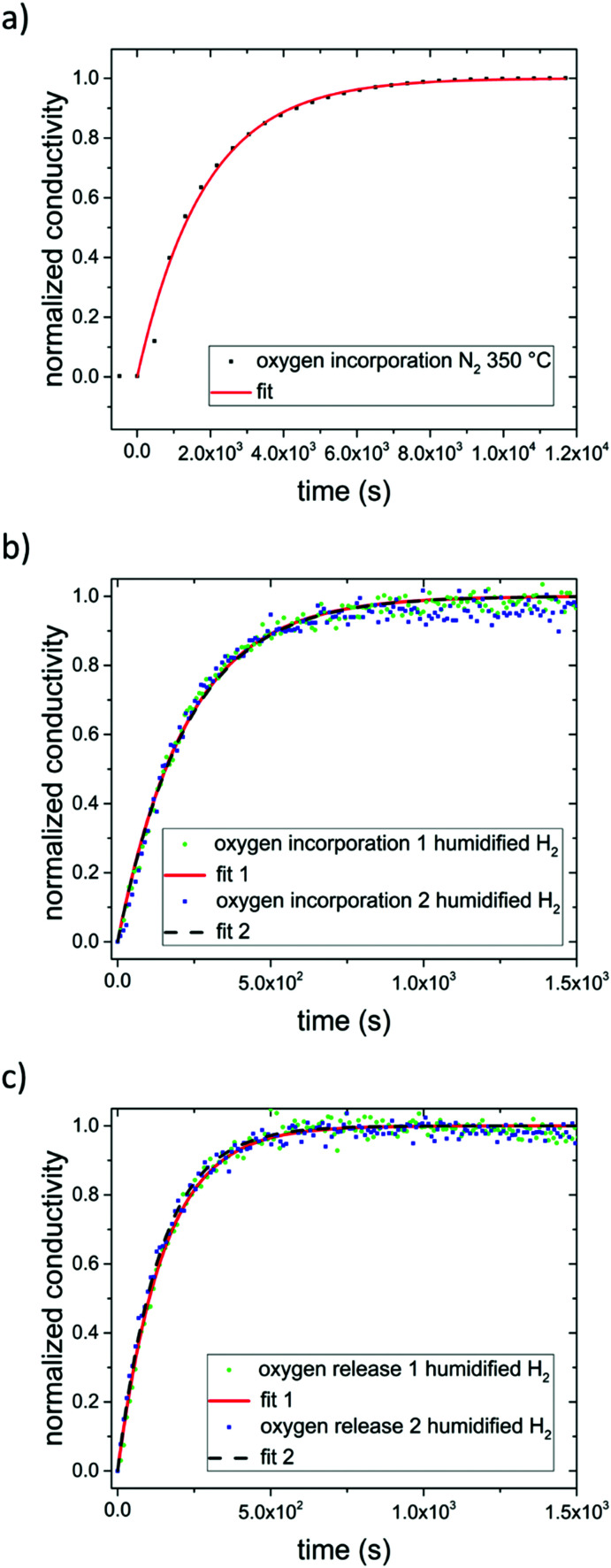
Normalized conductivity of SrTiO_3_ in N_2_ (a) and humidified H_2_ (b) at 350 °C under UV illumination. Fit to eqn (3) are represented by either a solid red line or a dashed black line. (c) Normalized conductivity of SrTiO_3_ after UV light was turned off in humidified H_2_ at 350 °C. Eqn (4) was used for fitting the experimental data.

Applying eqn (3) to our measurement data for N_2_ with some impurity O_2_ leads to a surface exchange coefficient of 2.7 × 10^−5^ cm s^−1^ for the oxygen incorporation under UV at 350 °C. The normalized data and fit are displayed in Fig. 10a. Without over-interpreting the fit, we can still state that such a transition of oxygen incorporation during UV illumination from diffusion limitation in air to surface limitation in N_2_ is in accordance with the fact of having much less oxygen available in the N_2_ gas phase. A lower limit of the oxygen diffusion coefficient can also be estimated since for an oxygen surface exchange limitation the oxygen chemical diffusion coefficient has to exceed at least *k*_chem_ × *a* × 10, here approx. *D*^*δ*^_O_ > 1.4 × 10^−5^ cm^2^ s^−1^. The relaxation of the conductivity after UV irradiation in N_2_ (and O_2_) is considerably slower than the conductivity enhancement under UV light, since the latter is based on oxygen incorporation which is accelerated by light. This is also in good agreement with kinetic studies on Fe-doped SrTiO_3_ upon UV illumination.^7^

In dry or humidified H_2_ atmosphere, however, the conductivity relaxation after UV irradiation is on a similar time scale of approx. 900 s (dry H_2_), or 600 s (humidified H_2_) respectively, as the changes under UV. Thus, the oxygen incorporation under UV irradiation and the oxygen release after UV are both limited either by diffusion or by the surface exchange kinetics are much faster than in air.^29–33^ At 350 °C in humidified H_2_ the fits using eqn (3) match the experimental data (see Fig. 10b). Hence, we suggest oxygen incorporation is limited by the surface reaction. A surface exchange coefficient *k*_chem_ of 2.2 × 10^−4^ cm s^−1^ was obtained which is about one order of magnitude higher than in N_2_. Using the same approximation as before *D*^*δ*^_O_ thus has to be at least 1.1 × 10^−4^ cm^2^ s^−1^. This is higher than the equilibrium diffusion coefficient of 9.4 × 10^−6^ cm^2^ s^−1^ predicted by our defect chemistry model of undoped SrTiO_3_ in humidified H_2_ and at 350 °C. This is only plausible, if UV irradiation leads to an increase of the oxygen chemical potential and effective *p*_O_2__ respectively (compare Fig. 7) since this also enhances *D*^*δ*^_O_.^24^ For example, the defect chemical model suggests 1.5 × 10^−4^ at 10^−25^ bar *p*_O_2__.^13^

In order to describe the oxygen release after the UV light was turned off, eqn (3) has to be slightly modified, since now both the front and the back side are to be considered in the process. Therefore, eqn (4) is introduced:4
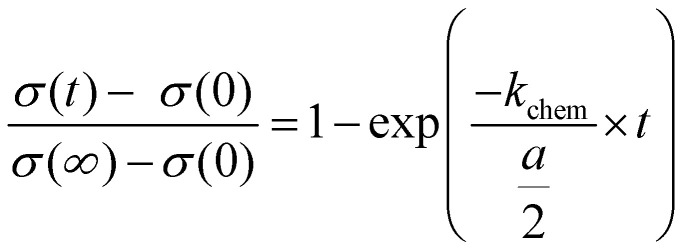


The fits (Fig. 10c) led to *k*_chem_ values of 1.7 × 10^−4^ and 1.8 × 10^−4^ cm s^−1^. This indicate that UV illumination alters the oxygen incorporation in humidified H_2_ only slightly.

Neither a model assuming diffusion or exchange limitation, nor a mixed regime model were able to describe the experimental data obtained from specimens contacted on both the front and back side properly. We suggest that the additional Pt coverage and the extended three phase boundary (SrTiO_3_/Pt/H_2_ atmosphere) may change in the oxygen exchange kinetics^10,34^ and thus also the diffusion profiles.^10^

### Model of the chemical processes under and after UV irradiation

Finally, we utilize all the consideration made to this point and introduce a model describing the defect chemical changes in SrTiO_3_ under and after UV illumination in a strongly reducing atmosphere (electrons are the major electronic charge carrier) at elevated temperatures. As SrTiO_3_ is a mixed conductor, it will adapt to the atmosphere *via* the oxygen chemical potential *μ*_O_. In the gas phase, *μ*_O_ is varying with partial pressure and temperature according to eqn (5).5
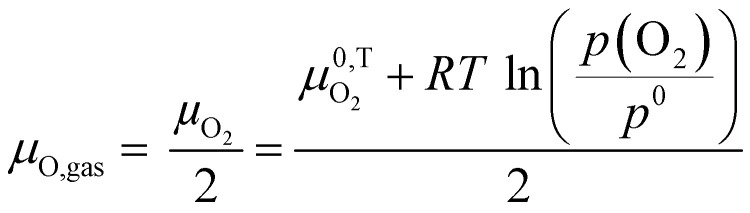


Even though there is no neutral oxygen present inside a mixed conducting oxide, the quantity *μ*_O_ can still be defined/calculated inside the material. The following equations show the different possible ways of calculating *μ*_O_ using either the chemical potentials of oxygen ions or oxygen vacancies and those of either electrons (eqn (6)) or holes (eqn (7)).^35–37^6
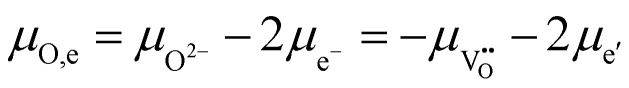
7
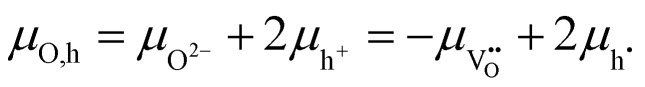


**Fig. 11:**
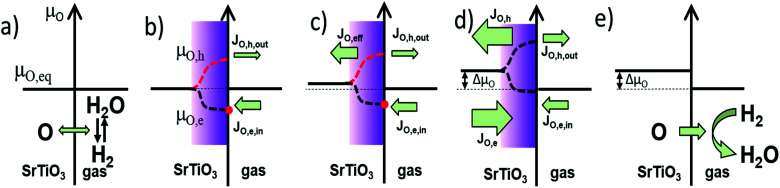
Model based on the formation of oxygen quasi-chemical potentials under UV illumination, explaining why oxygen is incorporated into and diffuses through an oxide-semiconductor, here SrTiO_3_. (a) Equilibrium oxygen chemical potential, oxygen incorporation into the semiconductor (by water splitting) equals the oxygen release into the gas phase (forming H_2_O). (b) As UV light is switched on, two oxygen quasi-chemical potentials are formed in the UV absorption zone due to the formation of electron–hole pairs. The dark bulk is not affected yet. The oxygen incorporation rate by conduction band electrons is enhanced. (c) In the UV absorption zone valence band hole connected oxygen chemical diffusion drives oxygen into the bulk. Oxygen diffusion in the bulk increases the oxygen chemical potential throughout the whole specimen. (d) A new steady state under UV illumination is finally established. Oxygen incorporation (*via* conduction band) is balanced by oxygen release (*via* valence band), the oxygen chemical potential in the dark bulk is increased by Δ*μ*_O_. (e) When the UV light is switched off, oxygen is released again out of the single crystal into the gas phase.

In equilibrium, the rates for oxygen incorporation and oxygen release are balanced, additionally the electronic chemical potentials for electrons and holes are linked (*via* the Fermi-level). Importantly, also the oxygen chemical potential *μ*_O_ is the same inside SrTiO_3_ and in the gas phase (compare Fig. 11a). This equalization of *μ*_O_ is the thermodynamic driving force behind the dependence of the oxygen nonstoichiometry of mixed conducting oxides on temperature and oxygen partial pressure.

When UV light is switched on, electron–hole pairs form inside the ∼3.2 eV bandgap semiconductor SrTiO_3_ thus splitting the Fermi-level into two electronic quasi-Fermi-levels for the now increased concentration of both electrons and holes – as is an established model for semiconductors.^38–40^ The same double increase of species alongside splitting into quasi chemical potentials is not possible for the ionic species (oxygen ions and oxygen vacancies) as their number is fixed by the lattice sites (3 per formula unit in ABO_3_ perovskites). Therefore, under illumination, the ionic chemical potentials in eqn (6) and (7) remain the same, but the electronic chemical potentials split. Consequently, also the values for *μ*_O_ split into different values in the illuminated zone of the SrTiO_3_ when either electrons (eqn (6)) or holes (eqn (7)) are considered.

This generates contradicting driving forces for oxygen exchange at the surface and for oxygen diffusion between the illuminated zone and the bulk, where SrTiO_3_ at first has the same *μ*_O_ value as the gas phase. As obviously no unified thermodynamic equilibrium can be established anymore, the kinetics of the respective reactions determine what steady state will be established and how. Two individual steady states are establishing at the surface (rates of oxygen incorporation *vs.* oxygen release) and at the transition from illuminated zone into the bulk (chemical diffusion of nominally neutral oxygen *via* combined diffusion of either oxygen vacancies plus electrons or oxygen vacancies plus holes).

Fig. 11 is a visualization of the kinetic situation that we encountered for undoped SrTiO_3_. As described above, it is only one possible steady state for an illuminated mixed conductor and one possible transient to reach it. Before illumination (Fig. 11a), SrTiO_3_ is in thermodynamic equilibrium with the atmosphere, and *μ*_O_ is equal in both phases, and eqn (5), (6), or eqn (7). Upon switching the UV light on, quasi-chemical potentials form in the illuminated zone for both electrons/holes as well as for *μ*_O,e_ and *μ*_O,h_ but the bulk is not affected yet (Fig. 11b). The hole-related oxygen chemical potential is higher than in the bulk and drives oxygen from the absorption zone into the bulk, while the electron related *μ*_O,e_ does the opposite. Empirically, we find an increased chemical potential in the bulk after UV and thus we suppose that the hole-related chemical diffusion coefficient is predominant in this “competition” (Fig. 11c). According to our measurements, we assume slow surface kinetics and fast diffusion, which leads to a temporary oxygen depletion in the UV absorption zone.

However, also oxygen incorporation (*via* water splitting) and oxygen release (*via* water formation from H_2_) are affected by UV due to the modified charge carrier concentrations. Oxygen incorporation rates due to water splitting are most probably enhanced by the additional photoelectrons in the conduction band and the same is true for oxygen evolution involving (photo)holes in the valence band. Empirically, we find a situation where under UV oxygen is continuously driven into the crystal. Hence, the electron related oxygen incorporation seems to be more accelerated by UV than the hole-related oxygen release (see Fig. 11b and c). The stronger enhanced electron related net incorporation flux (*J*_O,e,in_) warrants continuous oxygen supply to the UV zone and the bulk upon UV. Accordingly, the oxygen chemical potentials are successively pumped up inside SrTiO_3_. Finally, a steady state (not an equilibrium) is reached with equal diffusion fluxes and equal oxygen incorporation and evolution rates (Fig. 11d). The oxygen chemical potential in the bulk is increased by Δ*μ*_O_ compared to the chemical potential in the gas phase. When switching the UV light off, the oxygen quasi-chemical potentials vanish (Fig. 11f), and oxygen is slowly released back into the gas phase. Δ*μ*_O_ declines until equilibrium (*μ*_O,eq_) is reached again. A very similar qualitative description results for bulk diffusion limitation.

Please note that oxygen exchange *via* the non-illuminated planes were ignored in this model (as they are considered slow at the given temperature). Additionally, space charge effects were neglected. They may indeed play a role and would complicate the picture, however space charge effects are typically mitigated under UV light by the large number of photo generated charge carriers.

## Conclusion

Under strongly reducing H_2_-containing atmospheres UV irradiation of SrTiO_3_ led to a decrease in the in-plane conductivity at 350 °C. At the illuminated surface, an approx. 1 μm thick zone was established in which photoconductivity was two to three orders of magnitude higher than the bulk conductivity before UV irradiation. Nevertheless, in the remaining bulk the conductivity decreased gradually upon UV illumination, due to water splitting, subsequent oxygen incorporation and oxygen diffusion. The resulting effective *p*_O_2__ increase lowered the n-type conductivity of SrTiO_3_. For back side contacted single crystals, the resulting effects of the bulk conductivity shift were simulated by finite element calculations. Oxygen incorporation under and oxygen release after UV illumination seem both limited by the surface exchange reactions in humidified H_2_ and did not differ much. A change in the measurement configuration, where electrodes were placed at both the illuminated front side and the dark back side, led to a more pronounced photoconductivity effect which confirmed our interpretation, *i.e.*, the formation of a highly conductive region near the surface and a decreased bulk conductivity. Finally, the changes in the defect chemistry observed for UV illumination in H_2_ were explained by a model based on oxygen quasi-chemical potential. Mechanistically, UV illumination led to the same effects in air and N_2_, *i.e.* to a rise in the oxygen chemical potential. However, there the conductivity increased by orders of magnitude. This is in accordance with the defect chemistry of SrTiO_3_, since in the p-type conductive region an increased effective *p*_O_2__ means a higher conductivity.

## Conflicts of interest

There are no conflicts to declare.

## Supplementary Material
